# Multi-modal tomography to assess dechlorination treatments of iron-based archaeological artifacts

**DOI:** 10.1186/s40494-019-0266-x

**Published:** 2019-05-14

**Authors:** Mathieu Jacot-Guillarmod, Katharina Schmidt-Ott, David Mannes, Anders Kaestner, Eberhard Lehmann, Claire Gervais

**Affiliations:** 1 Bern University of the Arts, Fellerstrasse 11, 3027 Bern, Switzerland; 2 Collection Center, Swiss National Museum, Lindenmoosstrasse 1, 8910 Affoltern am Albis, Switzerland; 3 Paul Scherrer Institut, 5232 Villigen, Switzerland

**Keywords:** Dechlorination, Neutron tomography, X-ray tomography, Data fusion

## Abstract

Chloride ions are an important actor in the corrosion of iron-based archaeological artifact. To stop this degradation, excavated objects are subjected to dechlorination treatment. However, there is no guarantee that this will remove all chloride from the object, as some can be found deep inside the object. To assess the ability of dechlorination treatment to remove chloride, we propose to use both neutron and X-ray tomography. Indeed, these tomographic techniques have sensitivities to different elements and are thus complementary. Neutron tomography in particular is highly sensitive to the presence of chloride. This study demonstrate that this methodology allows to detect local and global changes caused by the dechlorination treatment, an useful tool to assess the effectiveness of a treatment and potentially improve it.

## Introduction

When buried in the ground, wrought iron archaeological artifacts are in contact with chloride ions residing in the soil. Once excavated from the soil, these chlorides, until then fairly passive, react with atmospheric oxygen and provoke an accelerated corrosion of the iron [1, 2]. One way to prevent this consists in submerging the metallic objects in liquid solutions designed to remove the chloride ions [3, 4]. Dechlorination treatments [5] stabilize the artifacts and stop the corrosion of freshly dug out objects. However, in some cases, objects whose corrosion has been stopped for years may resume a fast and strong corrosion [6] leading eventually to their complete destruction. This phenomenon is due to the presence of chloride ions lying deep inside the object that were not removed by the initial dechlorination treatment. After a prolonged period of time, those chloride ions can trigger a secondary corrosion process. It is thus crucial, for the long term efficiency of the dechlorination treatment, to assess the amount and the location of chloride present in the object. However, getting this information is not straightforward. Surface analysis techniques like Raman spectroscopy are useful to identify the corrosion products or to detect chlorides at the surface of the objects, but lack the ability to analyze the bulk of the objects in a non-destructive way. Tomographic techniques, while unable to identify materials as precisely, provide full 3D images of the objects making them potentially capable of detecting remaining chloride hidden deep inside the bulk. This is for instance the case of neutron tomography [7, 8]. Indeed, neutrons have a large cross-section for reacting through absorption and scattering with chloride atoms, so that the effectiveness of a dechlorination treatment can be evaluated rather easily by comparing images obtained before and after treatment [9].

X-ray tomography has been used to study many problems in the field of cultural heritage science [10, 11], but it is not able to assess the presence of chloride. Indeed, iron has a much higher absorption yield than chloride and the acquired scans would mostly indicate the concentration of iron. On the other hand, such information can help to interpret the neutron tomographic images by identifying the iron corrosion products, which have a lower attenuation compared to the pure iron core. Therefore, the comparison of both sets of images can be the key to locate both the iron corrosion products and the residual chloride ions inside archaeological iron-based artifacts [12, 13].

In this paper, we will demonstrate that the combined use of neutron and X-ray tomography is a powerful tool to investigate the effects of a dechlorination treatment. We apply this multi-modal tomographic approach to investigate roman iron nails found buried under the ground and assess the effects of their dechlorination treatment. For that purpose, we use bivariate histograms inspired by data fusion theory [14] to identify and localize the different types of materials within the nails. The characteristic attenuation coefficients of four reference powders containing pure iron corrosion products have been measured to help identifying them in the nails and analyze the impact of the treatment.

## Methodology

### Dechlorination treatment

**Fig. 1:**
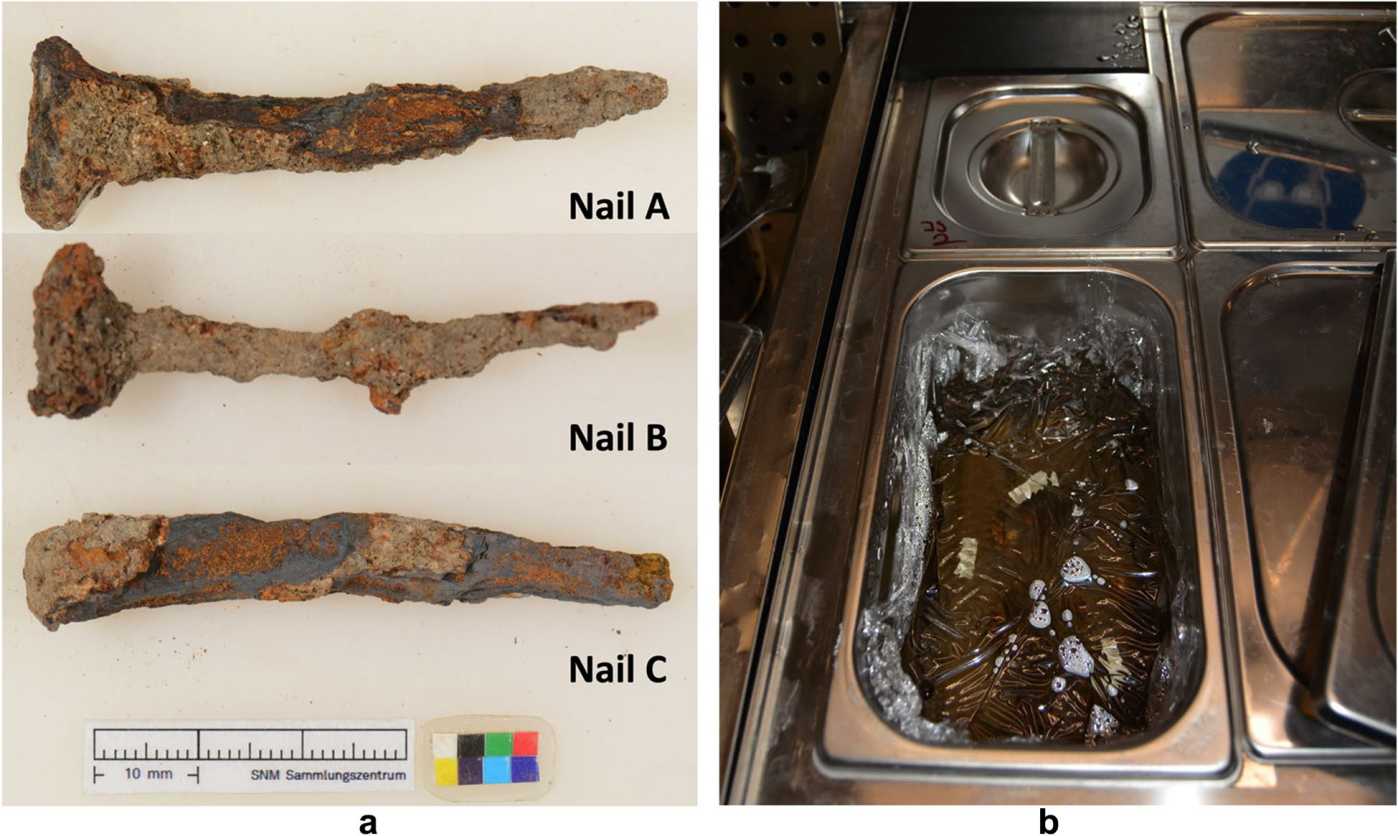
Roman nails used for the experiment and their dechlorination treatment performed at the Swiss National Museum (SNM).** a** Samples,** b** Dechlorination bath

Three nails (A, B and C) dating from the roman period were investigated (Fig. 1a). Nails B and C are treated using the dechlorination treatment described below [15–18]. Nail A stayed untreated to serve as reference and was conserved in a RP-A^®^ oxygen scavenger to avoid further deterioration.

The nails B and C were bathed in a solution made of demineralized water, 0.5 M sodium hydroxide (*NaOH*, 20 g/l) and 0.05 M sodium sulfite ($Na_{2}SO_{3}$, 6.3 g/l) (Fig. 1b). The sulfite ion (${SO_3}^{2-}$) serves as a reducing agent and neutralizes the dissolved oxygen by oxidation to produce sulfate. This process prevents the oxidation of iron in the dechlorination bath. Sodium sulfite is added to compensate for the consumption and the baths are covered with a polyethylene foil. The solution is alkaline with a pH value well above 12. In this range, Akaganéite ($\beta -FeOOH$) experiences a phase transformation and releases bounded chloride [19, 20].

The baths were changed every 16 days and four baths were used in total. The dechlorination is monitored by titration of the contents of the dechlorination baths. The treatment relies on the diffusion of chloride ions out of the iron-based artifacts and the chloride content is determined indirectly by titrating the amount of chloride ions in the washing baths (Aquamerck^®^ Chloride Test). The detection limit and error score of this technique lies by 0–5 ppm. When the measured chloride levels stabilize at a very low level, the process is stopped and the artifacts are rinsed with demineralized water and dried. In the present case, it took 9 weeks for the chloride levels of the bath to be below the detection limit.

Due to the high error rate of this measurement technique, only a rough estimation of the removed chloride can be made. For nail B ($m=12$ g), $\approx 26$ mg of chloride was removed and for nail C ($m=15$ g), $\approx 18$ mg was removed. At the end of the dechlorination process, all three nails including the reference nail were dried in a vacuum oven and then packed in silicagel conditioned to 8% RH to eliminate possible differences due to the drying process.

### Combined neutron/X-ray tomography

The neutron and X-ray tomography measurements were carried out at the neutron imaging instrument NEUTRA at the Paul Scherrer Institut (PSI) in Villigen (CH) [21]. Beside the possibility to perform neutron imaging experiments with thermal neutrons, the beamline is equipped with an optional X-ray source which can be used alternatively. Both options show similar beam geometry, which allows to directly compare the resulting neutron and X-ray data pixel by pixel [13].

As detector system, a scintillator-digital camera system was used. The neutron scintillator consisted of a $$ ^{6}{LiF{:}ZnS}{}$$-screen with a thickness of 50 μm, while for X-ray a CaWO OG16-screen was used [22]. As camera system, an Andor NEO sCMOS, with 2560 by 2160 pixels was used. The field of view was 123 mm by 104 mm and a corresponding pixel size of $$48 \times 48$$ μm^2^. The X-ray tube was operated at 160 kV and 9 mA with additional 2 mm Cu-filter. The computed tomography projections of the nails were acquired in 625 steps over $$360^{\circ }$$ with an exposure time of 45s per image for neutrons and 16s per image for X-ray, respectively. The projections were reconstructed into a three-dimensional image using the reconstruction software Octopus^TM^.

To prevent potential degradation during handling, the nails were enclosed in a sample-holder made of boron-free glass (which has a low attenuation coefficient for both neutron and X-ray). With this system, 4 images sets were acquired for each nail, 2 sets (neutron/X-ray) before and 2 sets after treatment.

### Image processing

Before comparison, the four 3D images (neutron/X-ray, before/after) of each nail were realigned through three-dimensional translation and rotations. To prevent physical damage, the nails could only be loosely fixed to the glass holder. This caused a displacement of the nail relatively to the holder between the measurements, which can lead to a faulty realignment. Before this alignment procedure, the glass holder was artificially removed from the images. For that purpose, a combination of filtering and segmentation was used to isolate the image of the sample-holder and remove this contribution from the unfiltered raw images. All image sets were then rescaled to the same resolution and cropped to obtain the same dimensions. This realignment was performed using the Avizo^TM^ software. Once this process is completed, all voxels with the same coordinates (*x*, *y*, *z*) correspond to the same volume of the nail for all four three-dimensional images.

### Multi-modal tomographic images analysis

The aim of the image processing procedure is to (1) detect the presence of chloride and (2) quantify the global and local changes of chloride amounts through the treatment. Segmentation of chloride-containing iron phases based on a single set of images is not appropriate here because X-ray images show a low sensitivity between iron-based material with and without chloride, while neutron images show the presence of chloride, but not of iron-based materials. Additionally, voxels could contain a mix of corrosion products with different concentrations of chloride and a segmentation would attribute each voxels to a material, meaning a loss of this information.

If the information provided by each tomographic technique taken separately is insufficient, their combination in a single bivariate histogram may lead to a clearer identification of the materials as function of their local amounts of chloride and iron. Bivariate histograms are conceptually similar to single modality histograms, but instead of transforming one single continuous parameter *i* into discrete one-dimensional bins of equal size to obtain a distribution $\rho (i)$, two independent continuous parameters $i_1$ and $i_2$ are transformed in two-dimensional bins to obtain a distribution $\rho (i_1,i_2)$. For this study, the parameters will be the attenuation coefficients for neutron ($\mu ^{n}$) and X-ray ($\mu ^{x}$) and the distribution will indicate the number of voxels with a specific combination of attenuation coefficients.

### Corrosion products identification

Chloride can be found as soluble ions or in the composition of corrosion products such as Akaganéite. Thus, it is important to identify the corrosion products to determine if the detected chlorides pose a threat to the material (soluble ions) or not (trapped inside the Akaganéite crystalline structure). As will be shown in the next session, the bivariate histograms show the presence of different phases, but do not allow to attribute them to a specific material. To determine where the different iron corrosion products are located in the bivariate histogram, X-ray and neutron images of four powder samples of pure corrosion products (Akaganéite, Goethite, Hematite and Magnetite) were acquired. These phases were selected because they are common phases found in this type of archaeological artifact. However, previous studies [1] have shown that Akaganéite, Goethite and Magnetite, but not Hematite, are results of the corrosion mechanisms related to the presence of chloride after excavation. Thus, while the presence of Hematite is not unexpected, the other corrosion products are more likely to be present.

The difference in the material density between these samples (powder) and within the nails (partly solid, partly powder) has to be accounted for. The ratio between neutron and X-ray linear attenuation coefficients remains however constant, since they are both proportional to the density. The values of the cross-section $\left( \mu / \rho \right)$ for neutron and X-ray are known.

1 $$\begin{aligned} \mu ^{n}_{\text {material}}& =  {} \left( \frac{\mu }{\rho } \right) _{\text {n}} \rho _{\text {material}} \end{aligned}$$

2 $$\begin{aligned} \mu ^{x}_{\text {material}} & =  {} \left( \frac{\mu }{\rho } \right) _{\text {x}} \rho _{\text {material}} \end{aligned}$$

3 $$\begin{aligned} \frac{\mu ^{n}_{\text {powder}}}{\mu ^{x}_{\text {powder}}} & =  {} \frac{\mu ^{n}_{\text {nail}}}{\mu ^{x}_{\text {nail}}} \end{aligned}$$

4 $$\begin{aligned} \mu ^{i}_{\text {nail}} & =  {} \frac{\rho _{\text {powder}}}{\rho _{\text {nail}}} \mu ^{i}_{\text {powder}} \quad \text { with } i = n,x \end{aligned}$$

These equations show that the attenuation coefficient of each corrosion product in the nail can be derived from the attenuation coefficient of the corrosion product in powder form, if the densities of both physical states ($\rho _{\text {powder}}$ and $\rho _{\text {nail}}$) are known.

## Results

### Visualization of local changes with neutron tomography

**Fig. 2:**
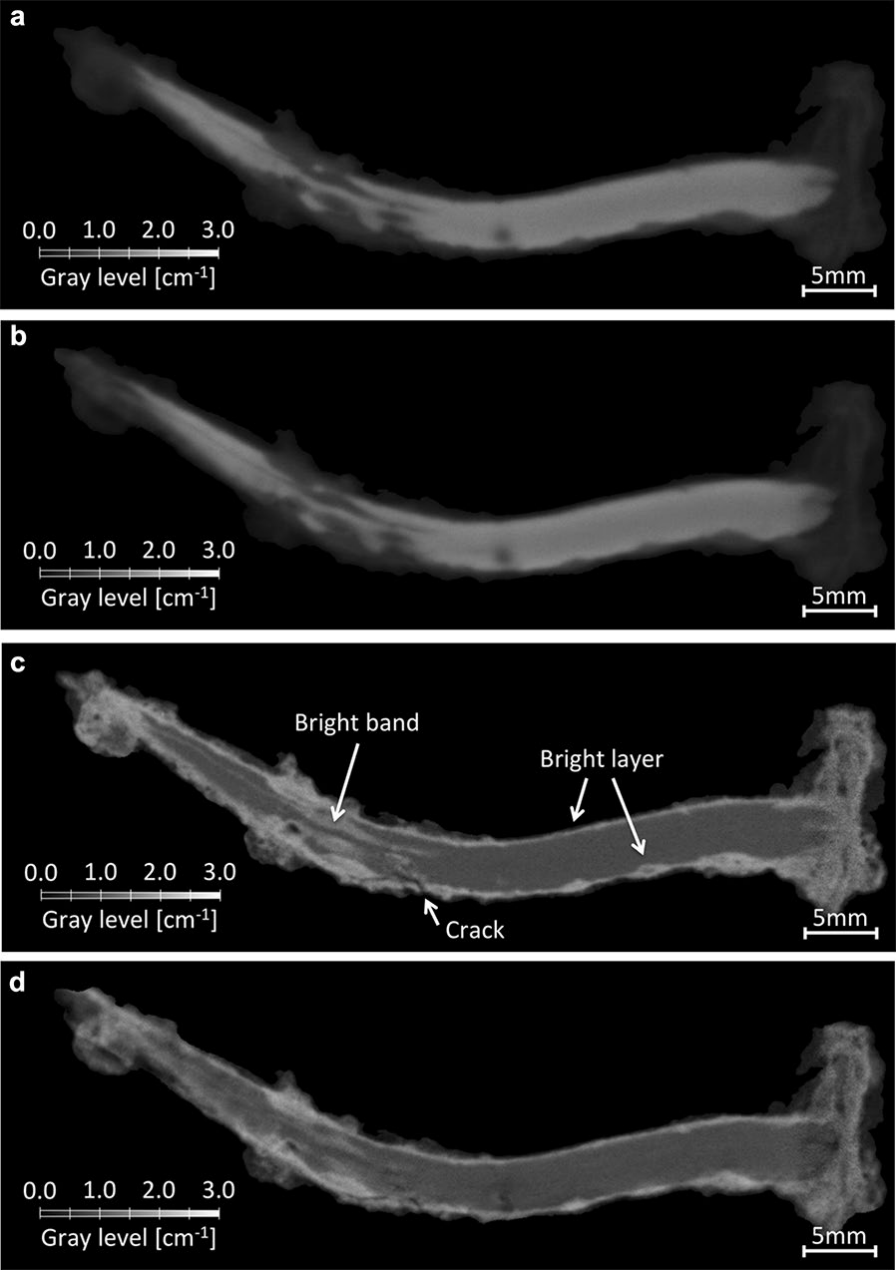
Tomography images obtained before and after treatment of nail B. There is no significant difference in the X-ray images (**a** and **b**), but some $\approx 100$ $\mu m$ wide bright band can be seen close to the core of the nail on the neutron images before the treatment (**c**), which disappear after treatment (**d**). Arrows indicate zones of interest.** a** X-ray tomography: before treatment,** b** X-ray tomography: after treatment,** c** Neutron tomography: before treatment,** d** Neutron tomography: after treatment

Figure 2 shows cross-sections of the raw 3D images of nail B. On the X-ray tomographic images, light gray levels correspond to the uncorroded iron core and dark gray to the corrosion products. The X-ray images do not show any remarkable difference before and after treatment. On the neutron tomographic images, corrosion products show up in bright (i.e. with a high attenuation coefficient) whereas uncorroded iron core appears in gray. The brightness level of the corrosion products are in agreement with the presence of a highly absorbing element such as chloride. Neutron images show also some changes between before and after treatment. The bright layer in the region close to the surface of the nails is thinner after dechlorination treatment. This is in agreement with a partial removal of chlorides in the corroded region through the treatment. It is also interesting to observe the interface between the head and the tip of the nail. There is some evidence of a structural weakness here. This could be due to the fabrication process, allowing the chlorides to infiltrate the nail deep inside the metal. On the neutron image acquired before treatment (Fig. 2c), this part is in light gray, indicating the presence of chloride. After treatment (Fig. 2d), this same region is in a gray darker than the uncorroded iron core. This phenomenon is not yet understood and will require further investigations as only iron or void is expected here.

A bright layer surrounding the core of the nail is observed in the neutron images before and after the treatment. The high neutron linear attenuation coefficients indicate the presence of chloride. If this assumption is correct, the absence of significant changes for these regions indicates that these chlorides were unaffected by the treatment. One explanation is the presence of chloride trapped inside the Akaganéite crystalline structure. It has been proven that the dechlorination treatment is not able to remove most of the chloride present in this form [23], but also that this type of chloride is not susceptible to participate to the corrosion process. While no conventional treatment can remove these chlorides, they are also harmless for the object.

**Fig. 3:**
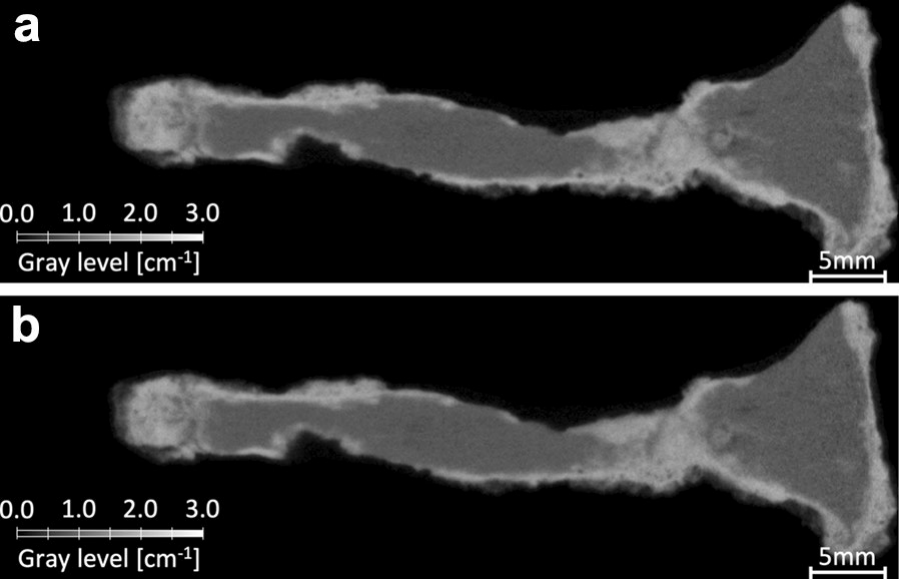
Tomography images obtained before and after treatment of nail A. While Nails B and C show clear difference between images before and after treatment, none significant are observed on the Nail A which was left untreated. This confirms that the changes observed in Nail B and C are caused by the treatment.** a** Neutron tomography: before treatment,** b** Neutron tomography: after treatment

As for the untreated nail A, a straightforward visual comparison shows no significant changes (Fig. 3). Thus, not only the observations made on Nail B are consistent with the expected effect of the treatment on the nail, but none are observed on an untreated nail. This demonstrates that the changes are very likely caused by the treatment and not some outside factor.

**Fig. 4:**
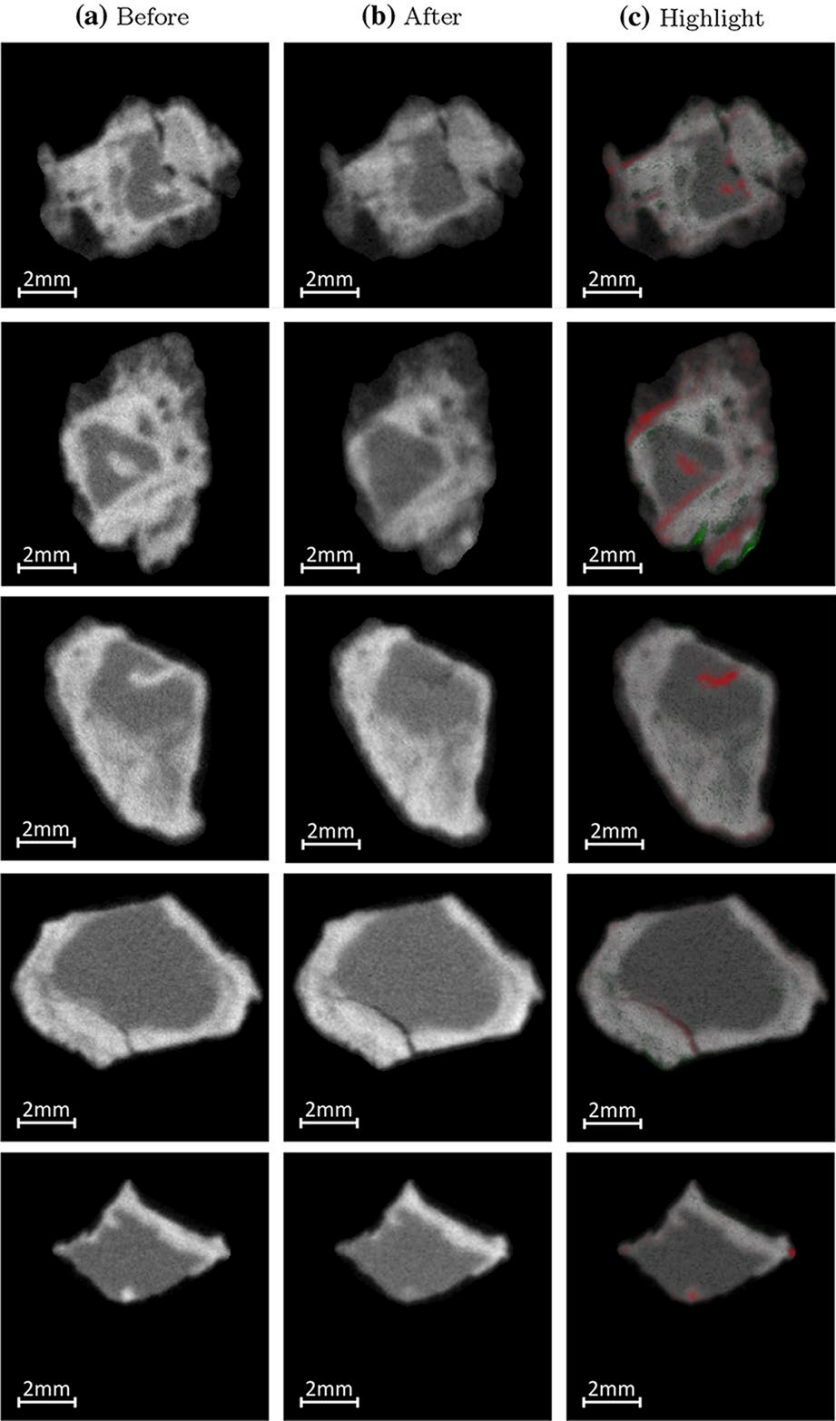
Neutron tomographic images of nails B and C before (**a**) and after (**b**) treatment in the plane perpendicular to the tip.** c** show a comparison by subtraction of the two images (**a**) and (**b**), overlapped on image (**b**), with decrease of intensity in neutrons in red and increase of intensity in neutrons in green.** a** Legend 1st column: before,** b** legend 2nd column: after,** c** legend 3rd column: highlight

Additional cross-section images in the plane perpendicular to the tip of the nails give more insights on the effects of the treatment on nails B and C (Fig. 4). Some bright bands ($\approx 100$ μm wide) appear before treatment on the neutron tomographic images. These bands are present deep inside the nails, probably indicating that the chloride ions have penetrated in the metallic structure using cracks or micro-porosity. Their complete disappearance on the neutron tomographic image after treatment indicates that the dechlorination process was able to remove them by infiltration of the dechlorination solution through the same porosity paths.

Other changes can be seen in the cracks ($\approx 10$ μm wide) of the nail, where the neutron attenuation decreases significantly. These fissures are usually present on the surface of the nails and are caused by the brittleness of the corrosion products. These cracks are often filled with residual soil material and possibly small fragments of corrosion products, which act as natural consolidators and hinder the further embrittlement of the corrosion product layer. Thus, the decrease in neutron intensity after treatment can be due either to the extraction of chloride by the treatment or to a removal of residual material in the fissure. In the latter case, it would lead to a weakening of the structural integrity of the object and should be mitigated or completely avoided. Higher resolution images may help to clarify which hypothesis is correct.

### Interpretation of the bivariate histograms

**Fig. 5:**
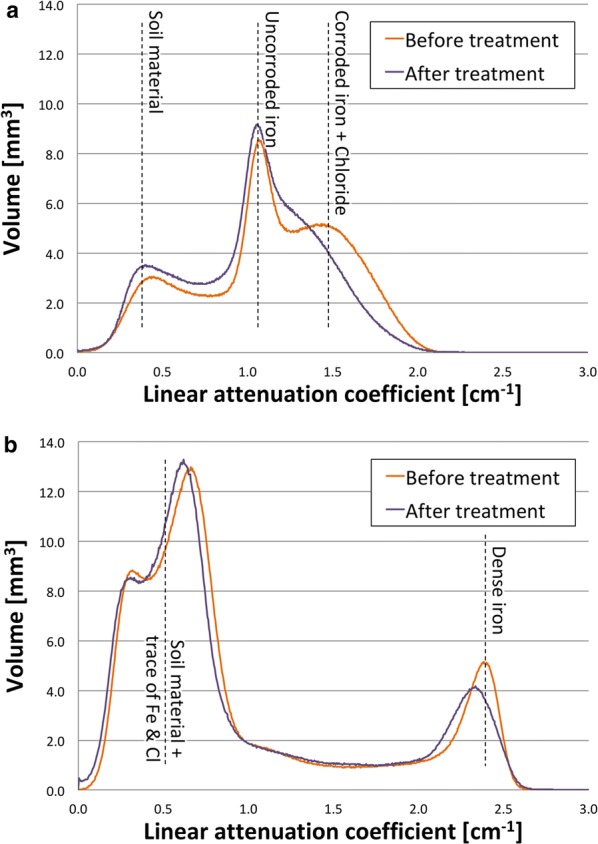
Histogram of linear attenuation coefficients for nail B before and after treatment. The histogram **a** shows a clear difference for higher attenuation values, corresponding to chloride-based material. The X-ray histogram shows no clear difference.** a** Neutron linear attenuation coefficient histogram,** b** X-ray linear attenuation coefficient histogram

Simple histograms of the image in neutron and X-ray show that, separately, they are not sufficient to identify the different materials (Fig. 5). Additionally, X-rays have no sensitivity to chloride and neutrons cannot differentiate between iron corrosion products. However, bivariate histograms are able to combine the advantage of both methods, while mitigating their flaws.

**Fig. 6:**
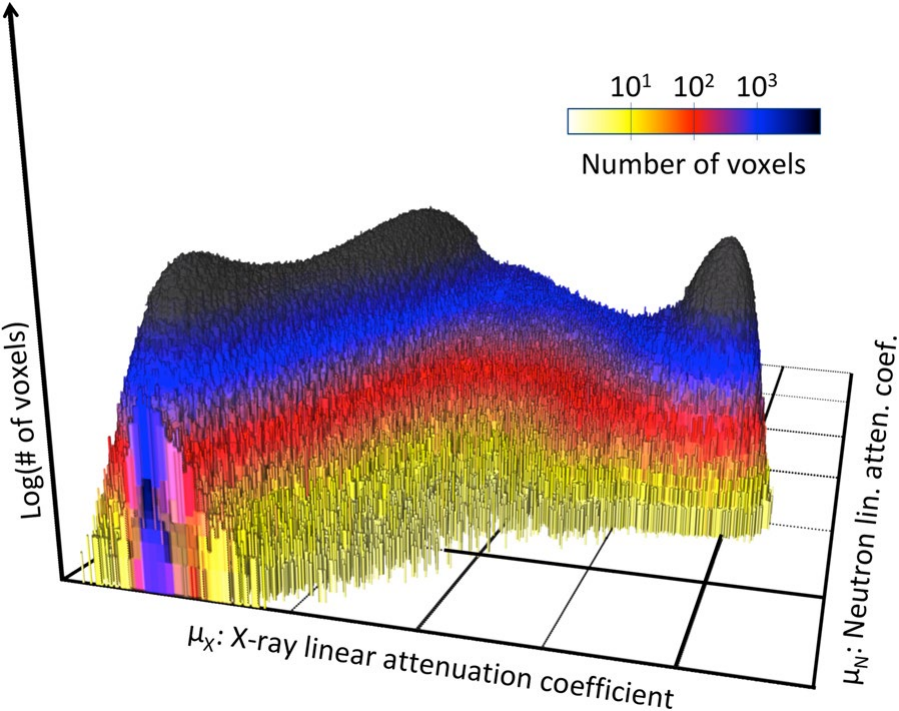
A surface plot of a bivariate histogram showing the abundance of each material with a specific pair of linear attenuation coefficients in neutrons and X-ray ($\rho (\mu ^{n},\mu ^{x})$) for nail B

**Fig. 7:**
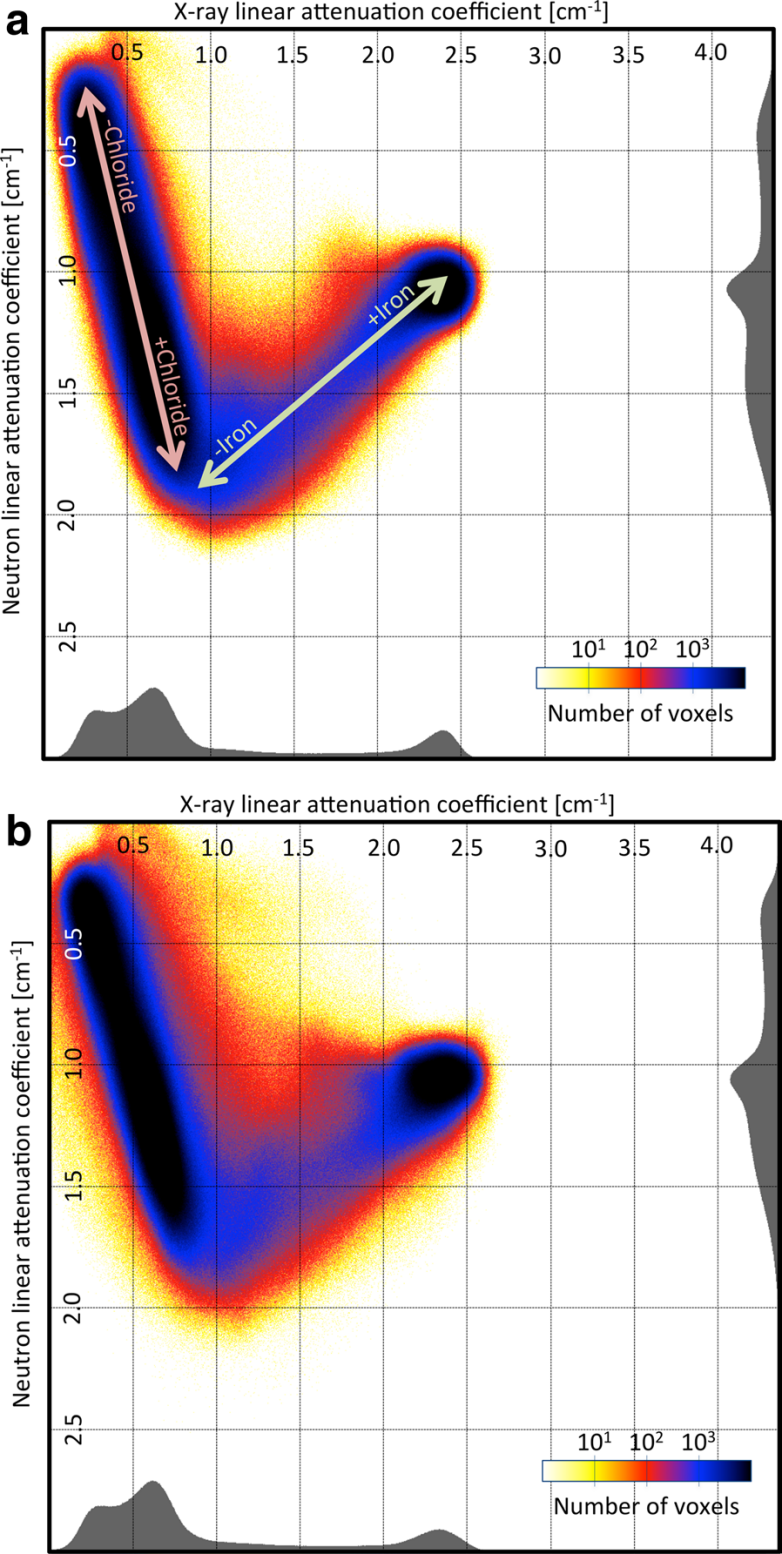
Bivariate histogram of nail B projected in the plan of the linear attenuation coefficient axes: neutrons vs X-ray before (**a**) and after (**b**) treatment. The histograms are distributed in two main axis that can be interpreted as gradients of concentration of chloride for the first and iron for the second. Most changes can be observed in a zone centered around $1.5$ cm^−1^ for X-ray and $1.5$cm^−1^ for neutrons.** a** Before treatment,** b** after treatment

The bivariate histogram for nail B is shown on Fig. 6. To allow for a better visualization, the number of events is color-coded on a logarithmic scale and projected on the $\mu ^{x}$, $\mu ^{n}$ plane (Fig. 7). The projection on the X-ray or neutron axis leads to a single modality histogram, shown in gray on the side.

Figure 7a also shows that most voxels are gathered along two main axes. The first axis goes from low to high neutron linear attenuation coefficient and from low to medium X-ray linear attenuation coefficient. This could be interpreted as an axis of chloride concentration, as an increase in chloride concentration would translate into a slight increase in X-ray and a large increase in neutron linear attenuation coefficient. The second axis goes from high to medium neutron linear attenuation coefficient and from medium to high X-ray linear attenuation coefficient. This could be interpreted as an axis of iron concentration, as an increase in iron concentration would translate into a large increase in X-ray. High iron concentration would also mean less porosity, thus less possibility for chloride to permeate the material, thus a decrease in neutron linear attenuation coefficient. The region (−iron) probably refers to iron corrosion products containing chlorides but with lower iron density, therefore lower X-ray attenuation capacity. The region (+iron) refers to uncorroded iron, with high iron density and no chloride.

### Corrosion products identification

**Fig. 8:**
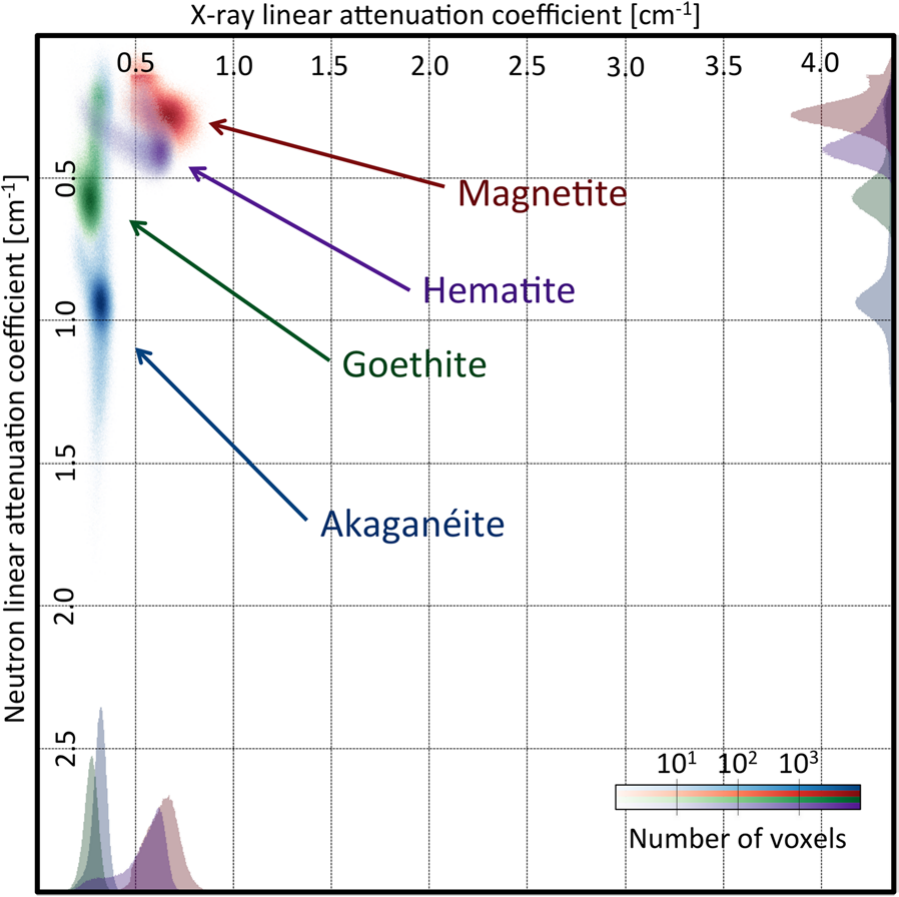
Bivariate histogram: reference corrosion product powders. 4 distinct regions are observed for the powders. They can not be reported directly in the bivariate histogram of the nail, because of the difference of density of the corrosion products in the powder and in the nail

To determine how the dechlorination treatment affects the different corrosion products, it is necessary to locate them in the $\mu ^{x}$, $\mu ^{n}$ space of the bivariate histogram. We used a bivariate histogram for that purpose, obtained from the four reference iron oxide powders (Fig. 8). Superposition and localization in the bivariate histogram of the nail is not possible with this data, because of the difference of density between the powders and the corrosion products in the nail. However, the proportion of the different elements in the corrosion products is known so that the average X-ray and neutron linear attenuation of the iron corrosion products in the powder $\langle \mu ^{n/x}_{\text {powder}} \rangle$ can be computed. Additionally, the powders are composed of pure corrosion products and, while their density is unknown, their linear attenuation coefficient can be directly computed from the image. Therefore, for each chemical element *i* contained in the corrosion products (CP):

5 $$\begin{aligned} \left( \frac{\mu }{\rho } \right) _{\text {CP}} = \frac{\sum _{i} m_{i} \left( \frac{\mu }{\rho } \right) _{i}}{\sum _{i} m_{i}} \end{aligned}$$

For each voxel *j* in the powder image is stored a value $\mu _{j,\text {powder}}$, similar, but slightly different from one voxel to another, due to the inconsistent density $\rho _{\text {j,powder}}$ of corrosion products found in each voxels.

6 $$\begin{aligned} \mu _{j,\text {powder}} = \rho _{\text {j,powder}} \left( \frac{\mu }{\rho } \right) _{CP} \end{aligned}$$

However, it is possible to recover the average density of a powder $\langle \rho _{\text {powder}} \rangle$ by averaging the $\mu _{j,\text {powder}}$ found on the image.

7 $$\begin{aligned} \langle \mu _{\text {powder}} \rangle = \langle \rho _{\text {powder}} \rangle \left( \frac{\mu }{\rho } \right) _{\text {CP}} \end{aligned}$$

The densities of the corrosion products in the nails are known. Thus the multiplication of the linear attenuation coefficient in the powder by the ratio of the density in the nails and powder allows for a direct comparison of the linear attenuation coefficients.

8 $$\begin{aligned} \mu _{j,\text {nail}}& =  {} \rho _{\text {CP,nail}} \left( \frac{\mu }{\rho } \right) _{j} = \frac{\rho _{\text {CP,nail}}}{\rho _{\text {CP,powder}}} \mu _{\text {j,powder}} \end{aligned}$$

9 $$\begin{aligned} \mu _{j,\text {nail}} & =  {} \left( \frac{\rho _{\text {CP,nail}} }{\langle \mu _{\text {powder}} \rangle } \left( \frac{\mu }{\rho } \right) _{\text {CP}} \right) \mu _{\text {j,powder}} \end{aligned}$$

**Fig. 9:**
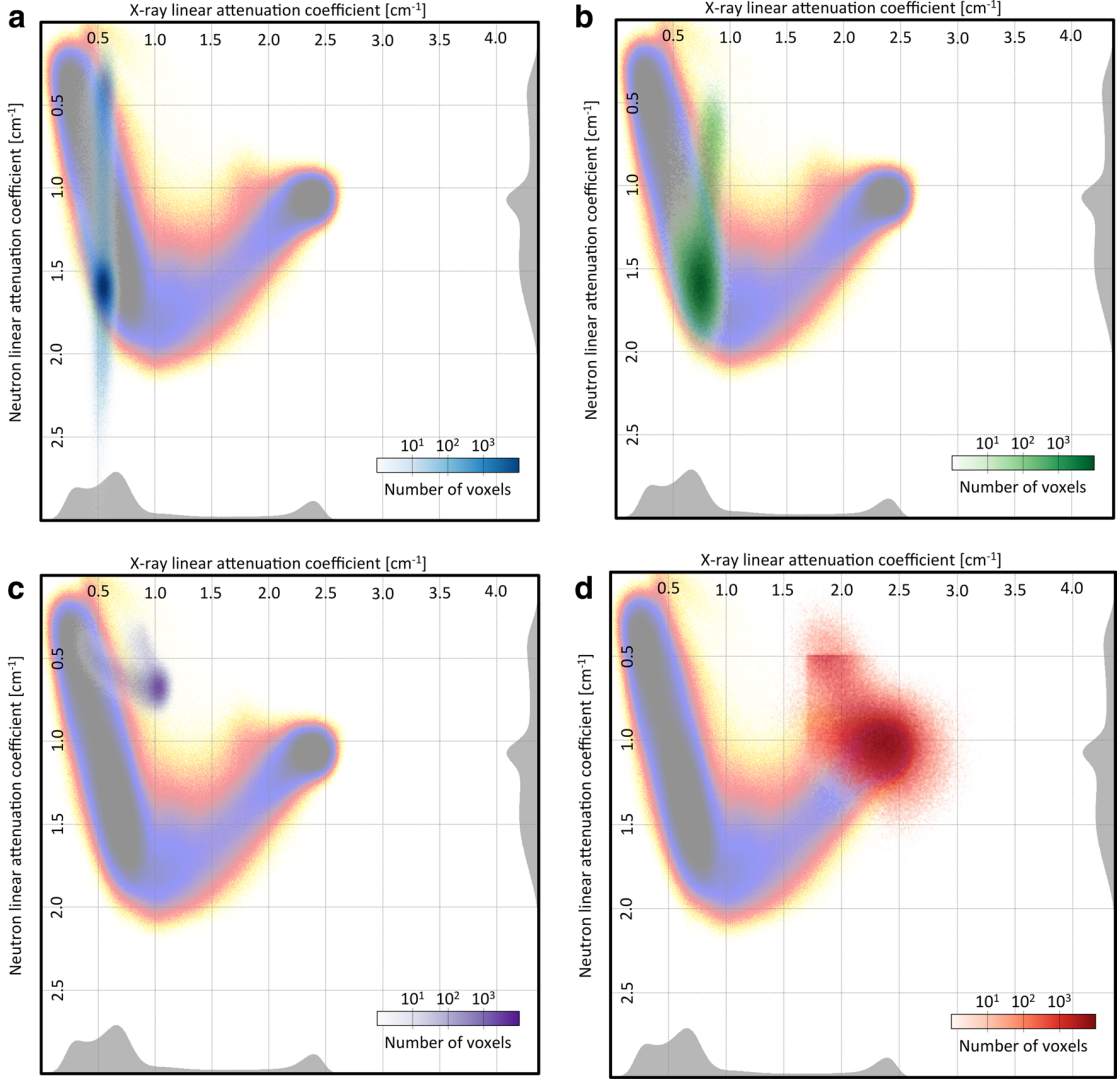
Bivariate histograms of the corrosion products powders after density correction, superposed with bivariate histogram of nail B.** a** Akaganéite,** b** Goethite,** c** Hematite,** d** Magnetite

Equation 9 shows that it is possible to obtain the linear attenuation coefficient of the corrosion products in the nail, from the linear attenuation coefficients of the corrosion products in the powder. Figure 9a–d show bivariate histograms of nail B superposed with a density-corrected bivariate histogram of the four reference corrosion products. For Akaganéite, Goethite and Magnetite, the bivariate histograms superpose well with the bivariate histogram of the nail. Akaganéite, containing chloride and hydrogen, both interacting strongly with neutrons, fills an area at the intersection between the chloride and iron axes. Goethite, with a 1:1 ratio between iron and hydrogen, also has a strong interaction with neutrons and covers an area close to Akaganéite. Magnetite, with no chloride and only iron and oxygen, has a bivariate histogram abundance zone centered very close to the uncorroded iron. For Hematite, no superposition was found. This can be due to the fact that no Hematite is present in the nail.

### Effects of the treatment on regions of the bivariate histogram

**Fig. 10:**
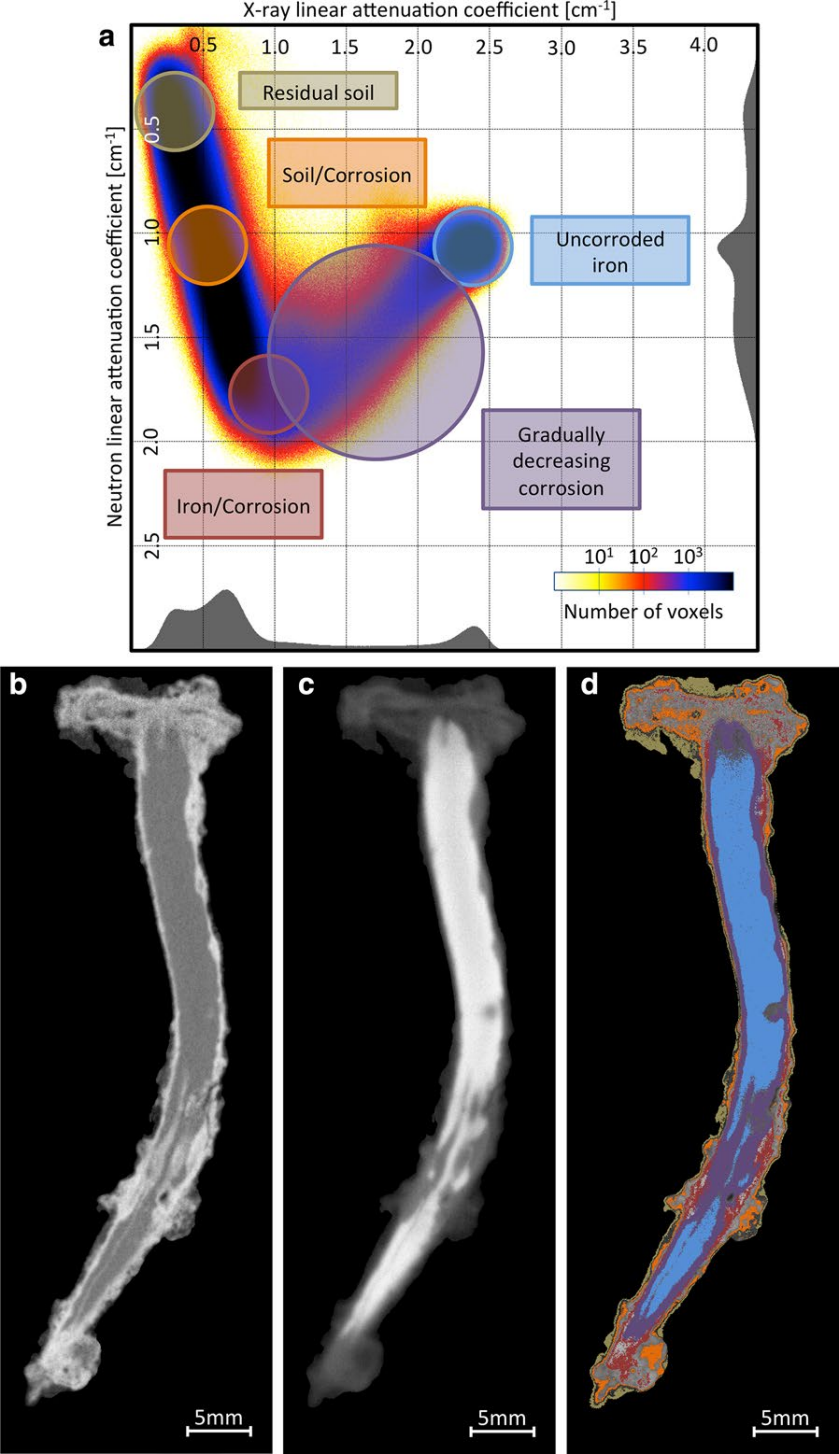
Spatial distribution of the materials found in predefined zones of the bivariate histogram of nail B. Using the bivariate histogram, it is possible to define regions of linear attenuation coefficients. Then, voxels with values of linear coefficients included in these regions can be found in the images, allowing to associate the regions in the bivariate histogram to material in the nails. Yellow corresponds to residual soil material, red corresponds to material with high chloride and/or high hydrogen density and orange corresponds to a mix of material. Blue is the uncorroded iron core. Violet is the zone of interest where the strongest effects of the treatment have been observed.** a** Bivariate histogram,** b** Neutron,** c** X-ray,** d** Phases

Locating the corrosion products in the bivariate histogram allows to visualize their spatial disposition inside the nail. In Fig. 10, we defined circular zones containing pairs of linear attenuation coefficients in neutrons and X-ray in a few zones of interest. The yellow zone corresponds to values of low neutron and X-ray attenuation coefficients and identifies residual soil material, which are localized, as expected, at the surface of the nail. Further down the chloride axis, the orange zone represents a mix of residual soil material and iron corrosion products. Iron corrosion products containing chlorides are located in the red zone at the intersection of the chloride and iron axes. The blue zone regroups all voxels corresponding to the uncorroded iron core.

**Fig. 11:**
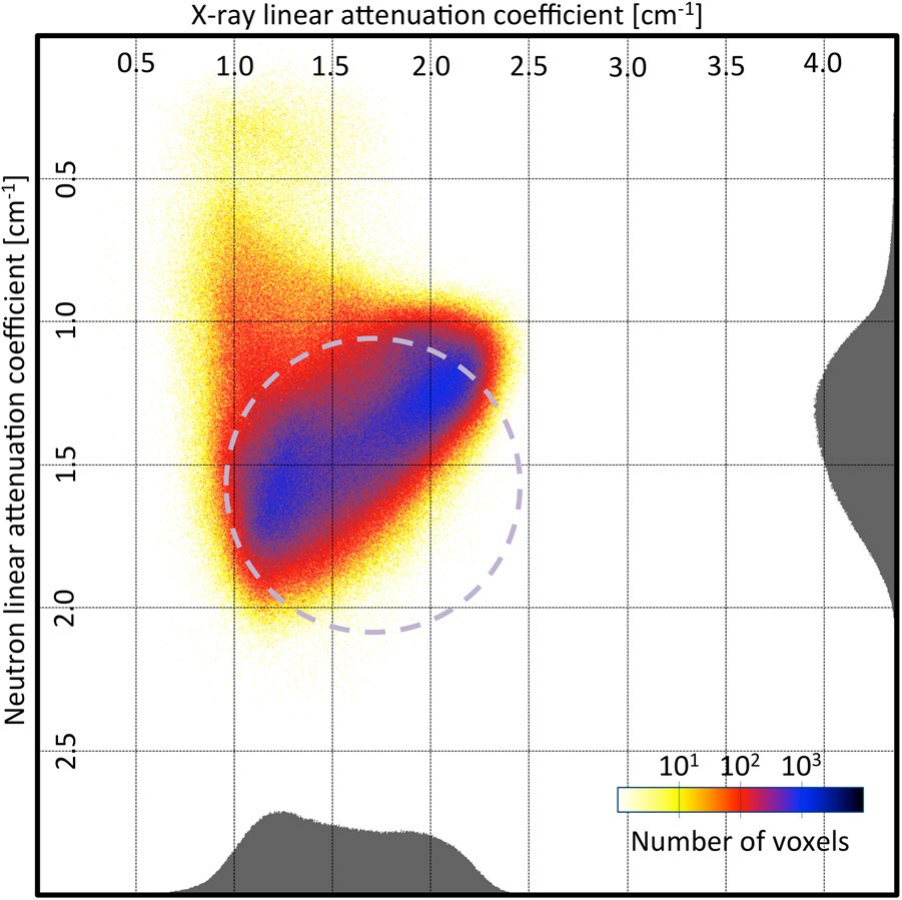
Bivariate histogram of the images after treatment of the violet zone defined in Fig. 10: Migration toward lower X-ray attenuation coefficient in X-ray for the zone of interest after the treatment

The violet zone should include iron mixed with chloride ions and therefore corrosion-prone. It is also where most of the changes before and after treatment are observed (Fig. 7). To visualize the effect of the treatment on the material included in this zone, we collected the coordinates of the voxels found in this region before treatment and produced a bivariate histogram for the values of attenuation coefficient $\rho (\mu ^{n},\mu ^{x})$ found in the images after treatment. Figure 11 shows that the material contained in the circle before treatment drifts toward lower neutron attenuation coefficient after treatment, while no significant change for the X-ray attenuation coefficient is observed. This is in agreement with a loss of chloride upon treatment in these regions.

One can also interpret this bivariate histogram like a population map, where materials migrate from one region to another through the treatment process. These movements can be quantified by defining the translation vector in the $\mu ^{x}$, $\mu ^{n}$ plane demonstrating the change in attenuation coefficients for a voxel before (BT) and after treatment (AT):

10 $$\begin{aligned} \overrightarrow{\Delta \mu } = \left( \begin{matrix} \mu ^{x}_{AT} - \mu ^{x}_{BT}\\ \mu ^{n}_{AT} - \mu ^{n}_{BT} \end{matrix} \right) \end{aligned}$$

**Fig. 12:**
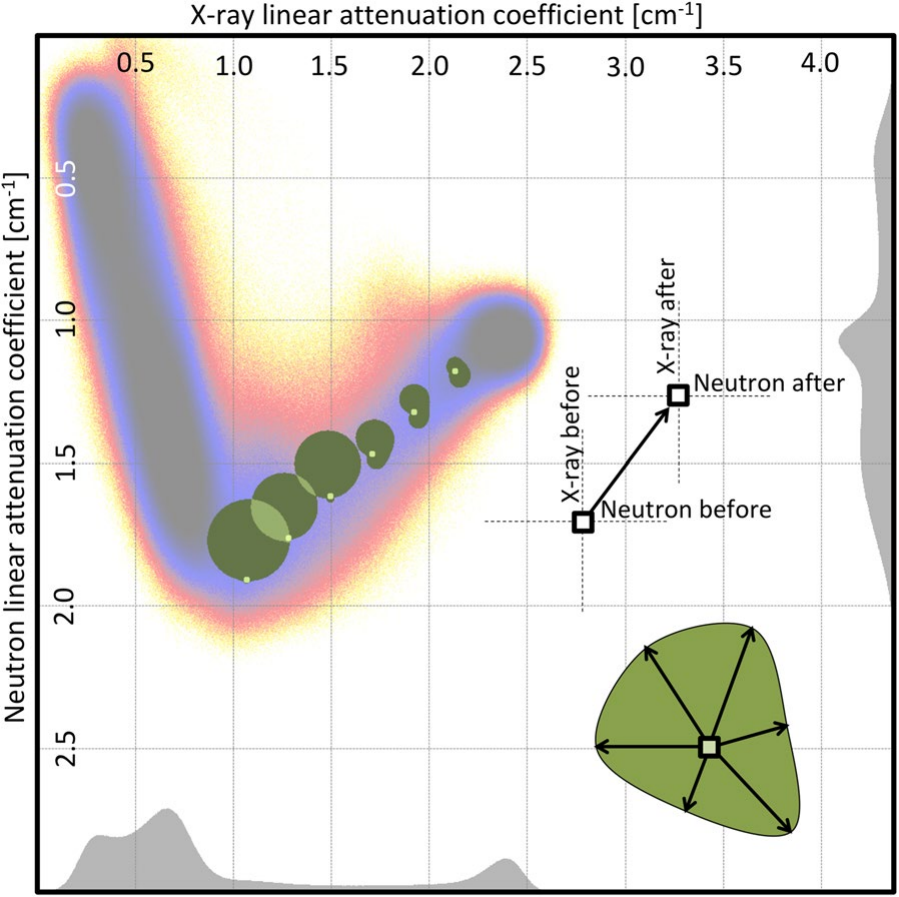
Hodograph: distribution of directional changes of attenuation coefficients. There is a clear directional change toward lower neutron attenuation coefficients for the zone at the start of the iron axis. Closer to the uncorroded iron, the distribution is more random

Using vectors, one can use common tools for migration modeling. Typically, what has been done previously for the violet zone can be applied on much smaller domains. Grouping all voxels with linear attenuation coefficients within an interval around attenuation coefficients $\mu ^{x}$ for X-ray and $\mu ^{n}$ for neutron, a vector defined as in Eq. 10 is computed and, from all these vectors, a directional distribution map, similar to a wind distribution map (also known as hodograph [24]), is obtained. On Fig. 12, a few of theses maps have been computed at different points along the iron axis. For low iron concentration, the changes are overwhelmingly directed toward a decrease of the neutron attenuation coefficients, while the change in X-ray attenuation coefficients is evenly distributed. For higher iron content, the hodographs do not show such directional trend and the changes are smaller. This can be interpreted as the result of the intrinsic noise of the images. To the contrary, those with a clear trend in their migration vector distribution indicate the domain of values of attenuation affected by the treatment.

### Estimation of the amount of chloride extracted

To further analyze the impact of the treatment on attenuation coefficients, an average movement vector $\left\langle \overrightarrow{\Delta \mu } \right\rangle$ is computed, using all voxels with linear attenuation coefficients within an interval around attenuation coefficients $\mu ^{x}$ for X-ray and $\mu ^{n}$ for neutron:

11 $$\begin{aligned} \left\langle \overrightarrow{\Delta \mu } \right\rangle & =  {} \left\langle \left( \begin{matrix} \mu ^{x}_{1} - \mu ^{x}_{0}\\ \mu ^{n}_{1} - \mu ^{n}_{0} \end{matrix} \right) \right\rangle \end{aligned}$$

12 $$\begin{aligned} \left\langle \overrightarrow{\Delta \mu } \right\rangle & =  {} \left\langle \overrightarrow{\mu }_{1} \right\rangle - \left\langle \overrightarrow{\mu }_{0} \right\rangle \end{aligned}$$

**Fig. 13:**
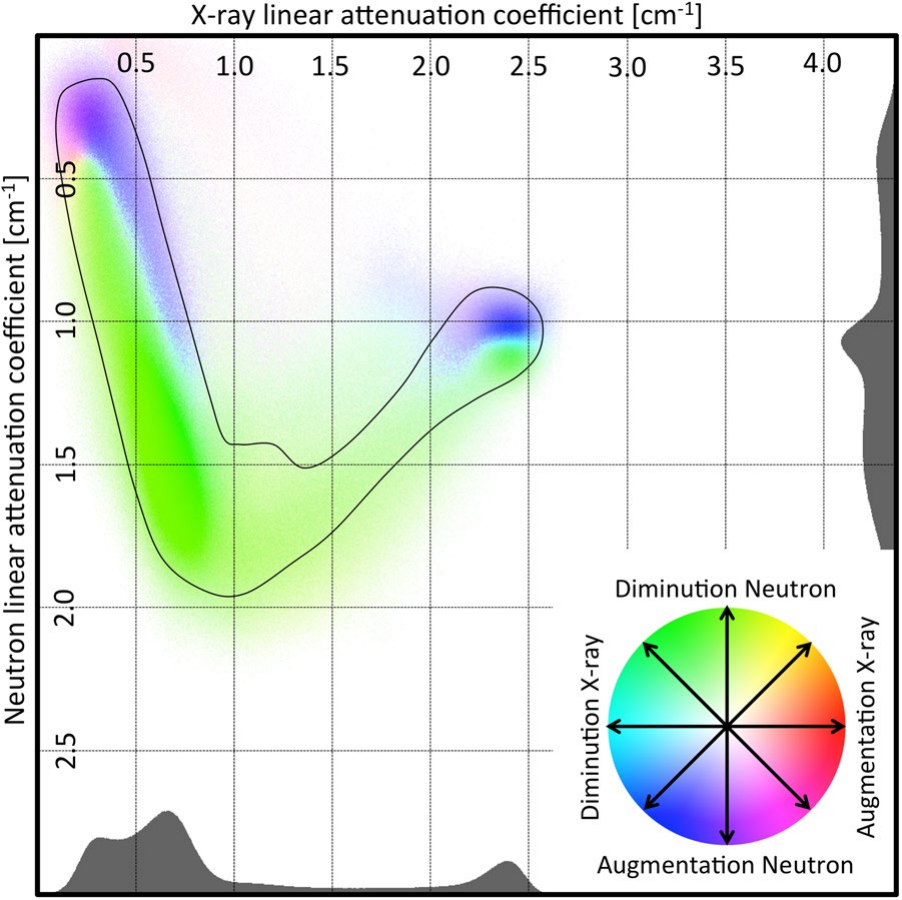
Direction map: average directional change in attenuation coefficient $\left\langle \Delta \mu \right\rangle$. The zone of interest clearly tends toward a decrease in neutron attenuation coefficients with no change in X-ray attenuation coefficients. The black envelop shows the border of the zone covering 99.9 % of the voxels

These vectors can be converted into a color corresponding to their direction, while the color intensity depends on the size of the vector. A direction map is generated accordingly (Fig. 13).

As addressed previously, most of the voxels on the bivariate histogram are regrouped around two axes and it can be assumed that it is only because of the imaging noise that they are not all located exactly on the axes themselves. When computing an average on a group of voxel corresponding to the same material, if the statistical sample is evenly distributed, $\left\langle \overrightarrow{\mu }_{1} \right\rangle$ should be located on one of the axes. Unfortunately, when computing $\left\langle \overrightarrow{\mu }_{0} \right\rangle$, the statistical sample is not evenly distributed because all $\overrightarrow{\mu }_{0}$ are equal to the same value and this leads to a coherent noise related effect that could hinder the interpretation of this kind of map.

To better understand the influence of this uneven statistical sampling, let’s imagine that the same imaging procedure that was applied on the nail was applied on a sample of an homogeneous material, unaffected by the treatment. The bivariate histograms before/after would be similar and show a two-dimensional bell curve centered around a point $\overrightarrow{{\mu}_{c}}$, corresponding to the linear attenuation coefficients of the material. The computation of $\left\langle \overrightarrow{\Delta \mu } \right\rangle$ for a point of the map $\overrightarrow{\mu }_{i}$ leads to:

13 $$\begin{aligned} \left\langle \overrightarrow{\mu }_{0} \right\rangle (\overrightarrow{\mu }_{i})= & {} \overrightarrow{\mu }_{i} \end{aligned}$$

14 $$\begin{aligned} \left\langle \overrightarrow{\mu }_{1} \right\rangle (\overrightarrow{\mu }_{i})= & {} \overrightarrow{\mu }_{c} \end{aligned}$$

15 $$\begin{aligned} \left\langle \overrightarrow{\Delta \mu } \right\rangle (\overrightarrow{\mu }_{i})= & {} \overrightarrow{\mu }_{c} - \overrightarrow{\mu }_{i} \end{aligned}$$

The Eq. 15 shows that the resulting vector for any point of the map is a vector going from the point to the center of the distribution. It means that, for a point of the map where the treatment has no effect, the resulting vector will be oriented toward the value of linear attenuation coefficient the corresponding material would have, if there was no noise. This is what is observed on numerous parts of the Fig. 13, especially in the region associated with the uncorroded iron core.

The direction map region associated with the chloride axis is dominated by two areas whose colors correspond to opposite directions, as is expected for a region dominated by noise related effects. However, the limit between the two zones should be located in the middle, if noise was the only reason for this disposition, which is clearly not the case. The green zone is larger than the blue zone, and given that the green corresponds to a diminution in neutron linear attenuation coefficient in a zone where chlorides are present, it is reasonable to conclude that, in this region, both noise related effects and treatment have a strong influence on the direction map. However, the magnitude of the noise effects does not allow to quantify this change.

While the direction map is dominated by this noise related effect, there is a region of the map which does not follow the general dynamic of having a vector directed toward the axes. The violet zone of Fig. 11 shows a diminution in neutron linear attenuation coefficient and no significant change to the X-ray linear attenuation coefficient (light green zone). This means that some phenomenon counterbalances the noise related effect and it is in agreement with a removal of chloride. This zone is well delimited and could be used to compute the overall chloride removed from the materials corresponding to this region. By summing the overall decrease of linear attenuation coefficient in this region and assuming that it is all caused by a removal of chlorine atoms, we estimate the amount of chloride removed at 53.2 mg for nail B.

## Discussion

### Impact of the dechlorination treatment and structure of the nail

As shown previously, we could demonstrate that neutron tomography is capable of visualizing changes due to the dechlorination treatment, in agreement with a loss of chlorine within the nails. The combination of X-ray and neutron images were very successful at checking the effectiveness of the dechlorination process by comparing the images obtained before and after the treatment and locating in the object regions where the chloride ions were removed. We identify three main features of interest.

#### Cracks

Figure 4 shows the chloride removal within the cracks through the treatment. At that stage, it is not possible to assess what process predominantly contributes to this removal: diffusion of the chloride outside of the cracks caused by the treatment or removal of the material filling the cracks by contact with water solution. In the latter case, the surface of the object would be weakened by the removal of this material that acts as a natural binder. This needs to be taken into account by conservators as this may contribute to a loss of structural integrity in the entire object. However, by allowing to detect significant changes in concentration of chloride in cracks with a width of about 10 micrometers, the neutron tomography shows its potential for investigating metallic archaeological artifacts.

#### Bright bands within the nails

The neutron tomographic images before treatment from Figs. 2 and 4 show the presence of bright bands with a width around $100$ μm, deep below the surface of nail B. Not only do these bands disappear after treatment, but they become darker than their surroundings made of non-corroded iron, and therefore show a lower neutron attenuation coefficient. This can be explained by the presence of semi-porous cavities where the chloride has slowly penetrated over centuries when the nail was buried. When chlorides are removed during treatment, they leave a void, showing low attenuation coefficients. There still remains to assess if all pockets containing chloride can be reached through the treatment. If not, it could explain the resuming corrosion after a certain amount of time. If, at one point in time, some cavities are accessible to the chlorides, but are sealed at a later point in time, either by new materials (corrosion products or residual soil material) or built-in pressure, the treatment would fail to get access to these cavities. Later, the cavities could be exposed once again, due to the relaxation of internal fissure or the creation of new cracks, triggering a second phase of the corrosion process.

#### Bright layer surrounding the core

No change is observed through the treatment for the bright layer surrounding the non-corroded iron interior of the nail (see Fig. 4). This layer is probably made of corrosion products containing chlorine atoms trapped inside, such as Akaganéite. This could explain why the dechlorination bath has no effect in this region. However, this type of chloride is not susceptible to participate to the corrosion process [23].

### Usefulness of bivariate histograms

One way of determining the efficiency of the dechlorination treatment is to quantify changes in elemental composition using three-dimensional imaging techniques. This study focuses only on the removal of chloride ions. Some changes observed on the images, such as the removal of soil material and small fragments of corroded material from the surface of the nail, are not relevant for this study, but were observed. Moreover, the difference in the noise component of the attenuation coefficients could be mistaken for a significant change caused by the treatment, while it is only a side effect of the imaging technique. As such, both material removal and noise contribution have a strong influence on the quality of the tomographic images and their evaluation. The challenge of this study was to design a method able to detect significant local changes in absorption coefficients and determine if it is caused by the removal of chloride ions, the removal of some other material or a side effect of the imaging process, i.e. either noise or other image artifacts. We have shown that the bivariate histograms representation is a powerful tool to match this challenge and leads to several interesting observations:

1. The use of tomographic images to generate the bivariate histograms from the neutron and X-ray attenuation coefficients makes it possible to demonstrate the presence of two main axes associated with the elemental concentrations of chloride and iron. By applying the same technique to four samples of reference powder corrosion products and comparing the histograms obtained to that of the nail (see Figs. 8 and 9), the local abundance of each type of corrosion product can be evaluated. This shows the possibility to associate zones of abundance in the plane $\mu _{x}$, $\mu _{n}$ to chemical elements and trace it back to their location within the nail. This procedure helps minimizing the loss of local information that would have resulted from a global approach using single histograms.
2. A comparison of bivariate histograms before and after treatment shows a clear difference in a coarsely defined area of the histogram. Relating this area to the corresponding physical part of the nail confirmed that it matches the 100 μm bright bands removed through the treatment. The corresponding voxels migrate in the bivariate histogram to a lower neutron linear attenuation coefficient. A comparison of the bivariate histograms using a vector approach leads to the generation of vector distributions (hodographs) (Fig. 12). A clear preferential direction is observed in the parts of the histogram corresponding to zones where the noise contribution can be neglected. A generalization of this approach to the whole histogram (Fig. 13), averaged and color-coded, shows a well-defined domain of linear attenuation coefficients strongly affected by the treatment.
3. Considering this zone, it was possible to compute the total shift in linear attenuation coefficient for neutrons. By using the tabulated attenuation cross-section, one can derive the total amount of chlorides removed from the iron-based part of the sample. For this nail, the amount of chloride removed is estimated at 53.2 mg. While this is a higher value than the total chloride amount measured in the dechlorination bath ($\approx 25.7$ mg), it is necessary to consider that the measurement from the dechlorination bath is imprecise and the estimated 53.2 mg consider all the chloride removed, meaning not only the chloride found in the bath, but also any amount that was removed during the rinsing and drying of the nails, as well as small pieces of sample, which fell down in between measurements. Thus, this value is in good agreement with the expected amount of chloride removed by the overall treatment.

### Treatment assessment

The ability to determine the amount of chloride removed from the object during treatment demonstrate the potential usefulness of bimodal imaging to assess the efficiency of dechlorination treatments. Nevertheless, this methodology needs to be developed further to access the critical information to determine the success of a treatment, the remaining amount of chloride in the object. Currently, if the effects of the dechlorination treatment can be easily detected, there is no method to differentiate chloride trapped in the corrosion products crystalline structure (thus inoffensive) and potentially harmful chloride ions. However, given the small amount of samples used for this study and the significant results obtained, a study made on an increased amount of samples with an imaging process specifically designed for this type of analysis, would certainly lead to progress in this direction.

Despite the current lack of information on the remaining chloride in an object, bimodal imaging can still be used to test treatment procedures. Numerous technical difficulties such as sample sizes or unavailability of the equipment leads to the impossibility to integrate tomographic techniques in everyday conservation-restoration procedures. However, it might be possible to use tomography on selected test samples to assess the effect of a dechlorination treatment, determine its weakness and potentially improve it. No precise estimation of the effectiveness of the treatment can be made, but the simple detection of bright bands within the nails after treatment (as shown on Fig. 4) is a clear visual indicator of the potential failure of a procedure.

## Conclusion

The design of dechlorination treatments to save archaeological artifacts faces a significant obstacle: it is currently impossible to non destructively quantify the effectiveness of different types of treatments, and therefore to compare them. The amount of chloride contained in the samples is unknown and each sample has its own characteristics that are difficult to compare from one another. Thus, the same treatment procedure could succeed to extract all chloride from one object, but fail for another. Furthermore, several consecutive treatments on the same object do not eliminate the possibility of a relapse of the corrosion process at a later time. In addition, the samples have historical value and successive procedures yield the risk of damaging the object. The use of phantom samples imitating closely the properties of artifacts buried for centuries in an environment with variable characteristics is currently impossible. Any innovative approach to conservation requires first and foremost the non-invasive detection of chloride ions.

We have shown here that combining X-ray and neutron tomography images allowed to get closer to a quantification of the effectiveness of a dechlorination treatments. The procedure, which consisted in generating bivariate histograms from X-ray and neutron data, provided some accurate information on the spatial distribution of the chloride in the sample. Furthermore, it allowed to determine what materials were affected by the treatment. Ultimately, it was possible to directly quantify the amount of chloride actually removed from the sample. This represents a huge advantage compared to actual methods that calculate this amount indirectly and are thus prone to bias. Beside, the visualization of successfully treated regions of the samples and those where chloride still remains after treatment offers an enormous advantage. Indeed, it allows to compare efficiently and optimize the parameters of the different treatment types. One limitation is that, given the equipment it requires, in particular for the neutron tomography, this procedure is not feasible for large-scale application or for large objects.

More generally, the imaging based tools developed and presented here have a much wider audience. The use of imaging techniques based on two fundamentally different kinds of interactions to investigate the complex composition of partially corroded material allows a very detailed analysis that would not be possible with a single imaging technique. The identification of chemical compounds using tomographic images with neutrons or X-rays separately is extremely difficult because of the lack of contrast for heavy or light elements respectively. With the bivariate histograms, it was possible to accurately locate the corrosion products using reference powders of typical corrosion products and by adjusting their density, to make them match the bivariate histogram of the whole object. By defining the areas of interest in the bivariate histogram, it was then possible to precisely locate and visualize the distribution of the corrosion products within the objects.

While neutron tomography delivers more valuable results than X-rays, the neutron images alone are not enough to obtain the same quality of results as those presented. The use of two sets of data as presented here showed to be very promising and a generalization to *n* data sets may yield even more promising outcomes. With only two data sets, we demonstrate that it already allows an in-depth analysis of a dechlorination treatment procedure. The same methodology should now be applied to objects treated with different dechlorination procedures. The data acquired with such study should lead to the development of improved treatments for iron-based archaeological artifacts.
